# Prompting with electronic checklist improves clinician performance in medical emergencies: a high-fidelity simulation study

**DOI:** 10.1186/s12245-018-0185-8

**Published:** 2018-04-27

**Authors:** Ronaldo Sevilla-Berrios, John C. O’Horo, Christopher N. Schmickl, Aysen Erdogan, Xiaomei Chen, Lisbeth Y. Garcia Arguello, Yue Dong, Oguz Kilickaya, Brain Pickering, Rahul Kashyap, Ognjen Gajic

**Affiliations:** 1 Department of Medicine, Division of Pulmonary and Critical Care Medicine Mayo Clinic, Rochester, MN USA; 2 METRIC, Mayo Clinic, Rochester, MN USA; 3 Department of Anesthesiology, Mayo Clinic, 200 1st St. SW, Rochester, MN 55905 USA; 4 Department of Anesthesiology and Reanimation, Suleyman Demirel University, Isparta, Turkey; 5 Department of Critical Care Medicine, Qilu Hospital of Shandong University, Shandong, China; 6 Department of Anesthesiology and Reanimation, Gulhane Medical Faculty, Ankara, Turkey

## Abstract

**Background:**

Inefficient processes of care delivery during acute resuscitation can compromise the “Golden Hour,” the time when quick interventions can rapidly determine the course of the patient’s outcome. Checklists have been shown to be an effective tool for standardizing care models. We developed a novel electronic tool, the Checklist for Early Recognition and Treatment of Acute Illness (CERTAIN) to facilitate standardized evaluation and treatment approach for acutely decompensating patients. The checklist was enforced by the use of a “prompter,” a team member separate from the leader who records and reviews pertinent CERTAIN algorithms and verbalizes these to the team. Our hypothesis was that the CERTAIN model, with the use of the tool and a prompter, can improve clinician performance and satisfaction in the evaluation of acute decompensating patients in a simulated environment.

**Methods:**

Volunteer clinicians with valid adult cardiac life support (ACLS) certification were invited to test the CERTAIN model in a high-fidelity simulation center. The first session was used to establish a baseline evaluation in a standard clinical resuscitation scenario. Each subject then underwent online training before returning to a simulation center for a live didactic lecture, software knowledge assessment, and practice scenarios. Each subject was then evaluated on a scenario with a similar content to the baseline. All subjects took a post-experience satisfaction survey. Video recordings of the pre-and post-test sessions were evaluated using a validated method by two blinded reviewers.

**Results:**

Eighteen clinicians completed baseline and post-education sessions. CERTAIN prompting was associated with reduced omissions of critical tasks (46 to 32%, *p* < 0.01) and 12 out of 14 general assessment tasks were completed in a more timely manner. The post-test survey indicated that 72% subjects felt better prepared during an emergency scenario using the CERTAIN model and 85% would want to be treated with the CERTAIN if they were critically ill.

**Conclusion:**

Prompting with electronic checklist improves clinicians’ performance and satisfaction when dealing with medical emergencies in high-fidelity simulation environment.

**Electronic supplementary material:**

The online version of this article (10.1186/s12245-018-0185-8) contains supplementary material, which is available to authorized users.

## Background

Acute critical illness is routinely treated by highly trained staff in specialized care units. However, the initial resuscitation, the “Golden Hour” (after the initial hour following physiological insult or trauma is most crucial for successful resuscitation) can be impeded by inefficient processes of care delivery [1]. A key factor contributing to this is that critically ill patients continually generate vast quantities of clinical data [2]. This information can overwhelm providers, especially those not specifically trained to work in fast-paced, high-stress environments [3]. It is crucial to develop strategies that streamline the processes of care to minimize clinical misjudgment [4].

Checklists are a validated tool for dealing with such challenges; long adopted by aviation and nuclear industries, they have recently been demonstrated invaluable in standardizing and improving clinical care [5]. Haynes et al. showed a decreased in complications and 30-day mortality after non-cardiac surgery by implementing a surgical safety checklist [6].

However, checklist implementation has been slow in many settings, often due to a perception that the tool disturbs existing workflows. One approach to reduce the disruption is the presence of an additional team member, a checklist “prompter” specifically tasked with ensuring checklist completion. This has been tested during ICU [7] rounds, and it was associated with decreased mortality and shorter length of stay [8].

Proper care and timely interventions are critical in initial resuscitation [1, 9–12] especially in situations with diverse provider background and training [13]. Algorithms like advance cardiac life support (ACLS) and advance trauma life supports (ATLS) have been created to structure the care provided in emergent cases. However, none of these algorithms address the more frequently encountered clinical problems like altered mental state, respiratory distress, syncope, and sepsis with a prescribed structured approach. Furthermore, these packages are based on memorizing and recall rather than providing easy access to informational cues to guide the resuscitation workflow.

It seems evident that providing an interface capable of summarizing this information and providing decision support and validated management algorithms would address a critical need [14]. We have recently designed and developed a novel electronic tool, the Checklist for Early Recognition and Treatment of Acute Illness (CERTAIN), to apply a standard approach to evaluation and management of the acutely decompensating patient which included process of care workflows and a designated prompter [8]. We hypothesized that a standardized approach to the evaluation and management of acutely decompensating patients using the CERTAIN model would improve clinicians’ performance and satisfaction in a simulated acute care environment.

## Methods

### Study participants

Participants were recruited from the trainees and staff of a tertiary care teaching medical center in Rochester Minnesota. Subjects included medical students, medical and surgical residents and fellows, nurse practitioners, and physicians. To ensure a common minimum level of competence and training, all volunteers were required to have ACLS/BLS certification to be able to participate. All subjects consented to being recorded and having their performance analyzed as part of this study and the Institutional Review Board approved the study protocol. Each participant attended two sessions, a baseline evaluation without the use of the CERTAIN and a second session with the CERTAIN after receiving training.

### Study setting

The study was performed at a high-fidelity multidisciplinary simulation center equipped with technologically advanced mannequins programmed to show complex findings and react just as a patient would to treatment decisions and with video/audio recording capabilities [15].

### Study design

**Fig. 1:**
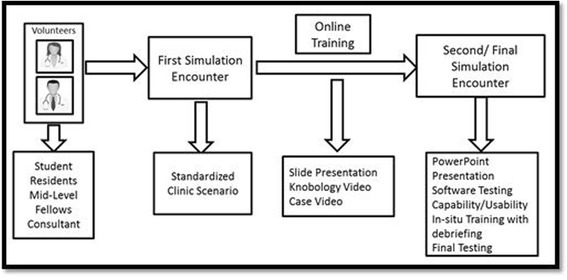
Graphic depiction of study design

Subjects came to the simulation center in groups of two or three for baseline testing. During the first session, the subjects would be fully oriented to the simulation center, the mannequin’s capabilities, and the study goals and expectations. After that, each was asked to perform evaluation and treatment of a simulated patient based on a standard 10-min clinical scenario representing close to 30 min of “real-time” action. During the scenario, two members of the research team would act as confederates, playing the role of nurse, respiratory therapist, or other resuscitation staff as needed. This study staff would perform actions such as starting IVs, obtaining labs or studies, and providing background as needed. In this role, they were instructed to act only on commands given by the team lead, not taking independent decisions on their own. To enhance the fidelity of the simulation, standard delays were part of each diagnostic test, e.g., laboratory results would be made available 2 min after the provider requested to reflect point-of-care laboratory testing. All encounters were videotaped, and later, they were scored based on the proportion of critical tasks completed in that particular scenario. Participants were provided with didactic material to be reviewed on their individual time consisting of a presentation describing the CERTAIN method and work flow, “knobology” video that described how to use the CERTAIN software, and an example video of a simulation case where research personal modeled the used of the CERTAIN model on a standardized clinical scenario. They were also provided with access to the CERTAIN software for their own independent practice in order to get familiarized with the use of the tool (Fig. 1).

Participants were asked to come back to the simulation center for a final session in groups of two to three clinicians in a minimum of 2 weeks for “washout” time. These 90-min sessions consisted of a brief 20-min didactic session, where we reviewed the most important components of the CERTAIN methodology and use, followed by a practical examination. The practical examination evaluated the participant’s ability to navigate the tool before allowing hands on the use of the CERTAIN in the simulator. A minimum passing grade of 80% was required before moving on. The participants were then allowed to practice with two to three nongraded scenarios to experience the team leader and prompter roles. A debrief period of 5 to 10 min was done after each practice session in order to give positive or corrective feedback as indicated. A final videotaped testing scenario was then evaluated, where the scenario was designed to reflect the same critical tasks as the baseline evaluation for that subject. Each scenario was designed to have two variations with similar clinical progression and scorable points but disguised with a different clinical vignette to minimize recall bias (see Additional files 1 and 2). Even though the clinicians were given feedback for usability, the actual testing components were not revealed to them at any point, prior to the final test. Ultimately, the participants were asked to complete an online survey regarding their opinion of the CERTAIN method on clinical practice [16] (see Additional files 3 and 4).

### Statistical analysis

All video recordings were evaluated by two independent reviewers. In cases of disagreement, the reviewers had the opportunity to replay the video and clarify based on discussion. However, if disagreement persisted, a senior critical care physician, using the definition on the SOP, would review the video and adjudicate disagreements. Each scorable item was graded as either “done” or “not done,” and the time from simulation start to item completion was recorded. The data were analyzed with the use of JMP statistical software (JMP Version 7. SAS institute Inc. Cary, NC, 1989–2012). All reported *P* values are two-sided, and *P* values of less than 0.05 were considered to indicate statistical significance. Each subject served as its own control, so paired comparisons using McNemar and Wilcoxon signed rank test were used as appropriate. Pooling data analysis of total task, specific, and generic data was also presented as indication of group performance.

## Results

From 24 participants enrolled in the simulation study, 18 completed both baseline and follow-up assessments; 11 critical care fellows, two residents, two visiting clinicians, and three medical students. Two thirds of the study participants were clinicians with at least 6 months of formal critical care training and were considered with high level of expertise.

Prompting with the CERTAIN effectively decreased the number omissions in both general (15 vs 29%, *P* < 0.01) and scenario-specific tasks (42 vs 59%, *P*< 0.01). The proportion of individual task completion is presented in Table 1. The overall pooled analysis of task completion was higher using the CERTAIN process, with omissions dropping by approximately one third (46 vs 32%, *P*< 0.01).

**Table 1:** Proportion of individual task completion with and without CERTAIN prompting

Item	Without CERTAIN (*N* = 13)	With CERTAIN (*N* = 18)	*P* value
Code status discussion	7 (39%)	12 (67%)	0.09
Airway assessment	10 (*56%*)	18 (100%)	*< 0.01
Breathing assessment	18 (100%)	18 (100%)	0.99
Cardiac assessment	18 (100%)	18 (100%)	0.99
Disability assessment	14 (78%)	13 (72%)	0.50
Exposure assessment	9 (50%)	15 (83%)	0.05
Evaluation of vital sign	18 (100%)	18 (100%)	0.09
Evaluation of temperature	10 (*56%*)	13 (72%)	0.23
Review of past medical history	12 (67%)	12 (67%)	0.62
Review of home medication	7 (39%)	11 (61%)	0.14
Review of allergies	8 (44%)	16 (89%)	*< 0.01
Order initial basic lab test	17 (94%)	18 (100%)	0.50
Start oxygen supplementation	18 (100%)	18 (100%)	0.99
Review of differential diagnosis	14 (78%)	15 (83%)	0.50
Total omissions (mean ± SD)	13 ± 4.3 (71%)	15 ± 2.7 (85%)	*0.01

*Statstically signficant *p*-value

Prompting with the CERTAIN leads to faster completion rate in most key assessment tasks (12 out of 14), suggesting a more efficient care on the group using the tool (Table 2). In the subgroup analysis by level of previous training, both groups (novice and expert) had similar improvement on the general tasks (expert *n* = 11, 3.5 vs 1.8 task omissions, *P* < 0.01; novice *n* = 7, 4.9 vs 2.4 task omissions, *P* = 0.03).

**Table 2:** Time to task completion on those cases that have task completion on the pre- and post-intervention test

Item	Number of cases available for assessment	Mean time change in seconds	*P* value
Code status discussion	5	− 181	0.04
Airway assessment	10	− 114	0.07
Breathing assessment	18	− 41	0.16
Cardiac assessment	18	− 92	0.05
Disability assessment	11	− 166	0.16
Exposure assessment	7	51	0.39
Evaluation of vital sign	18	− 31	0.56
Evaluation of temperature	8	− 73	0.35
Review of past medical history	7	51	0.34
Review of home medication	5	− 91	0.31
Review of allergies	8	− 5	0.92
Order initial basic lab test	17	− 79	*< 0.01
Start oxygen supplementation	18	− 53	0.27
Review of differential diagnosis	13	− 49	0.47
Total time to task completion	11.7 ± 5.2	− 62.4 ± 68	*< 0.01

*Statstically signficant *p*-value

Upon completion of their participation on the clinical scenarios, the volunteers completed a survey regarding their impression using the CERTAIN method to evaluate and treat critical ill patients. Seventy-two percent of them felt well prepared (four or more on a 5-point scale) when using the CERTAIN model. Eighty-three percent indicated that they would want to be treated by the CERTAIN model if they were critically ill. However, only one third (six of 18) of the subjects thought the software was easy to use (see Table 3).

**Table 3:** Survey result

Survey statement	Response score (*N* = 18)	Percentage of score with four points or higher (%)
The CERTAIN approach helps you feel better prepared during the emergency scenario	4.2 ± 1.0	72
The CERTAIN software was easy to use	2.7 ± 1.3	33
I would want to be treated by CERTAIN approach if I were critically ill or injured	4.2 ± 0.9	83
I think that checklist are useful in a medical emergency	4 ± 0.9	67

## Discussion

In a high-fidelity simulation environment prompting with the CERTAIN improved clinical task completion and decreased omission rates of critical tasks. Clinician satisfaction was high, with majority of participants wanting to incorporate this method to their own clinical practice.

Traditionally, resuscitation teams have been formed and trained to perform under stressful situations where they have to rapidly coordinate evaluation and treatment efforts [17, 18]. However, they have been structured around specific types of illness like trauma or cardiac arrest and largely rely on memory recall [18, 19]. Our experience demonstrated the performance of clinicians under stress is suboptimal at best, with an overall task completion rate of 50% at baseline. This is consistent with the findings of Smith et al. showing a decline in skill retention and loss of ability to perform ACLS and BLS skills to standard level when re-tested at 12 months [20].

As a potential solution, other studies have evaluated memory aids to improve health team performance in other situations. Haynes et al. tested the use of a checklist applied to regular operating room workflow on elective surgeries. His work showed a decrease on preventable surgical-related complications in the operating room [6]. Two studies designed for emergency teams dealing with late-phase resuscitation, one with a smart phone application [21] and the other a traditional checklist [22], had promising results in simulated environments. However, their designs did not include a prompter, which likely reduced their team’s compliance. Prompting with the CERTAIN is aimed to approach critical illness resuscitation earlier in the natural course of the disease, targeting to standardize the care on the so-called Golden hour. Early structured treatment has been shown to give better outcomes in simulated operating room crises [16], sepsis [23], myocardial infarction [24], and other critical illness states and providing a unified approach to decompensation may prevent the need of cardiopulmonary resuscitation efforts.

In a different setting, Weiss et al. tested the usefulness of prompting in critical care practice by implementing the use of checklist with a prompter versus checklist alone during daily ICU rounds. This study showed improvement in compliance with process of care, decreased length of stay, and a decrease in mortality [7]. These findings clearly demonstrate that important role prompting can play in facilitating complex process of care. The present study differs, however, in using a prompter in a higher stress environment with simulated acute medical emergencies. This stress imposes an extra burden on providers which could increase the risk of task omission. In this sense, the choreography of the CERTAIN with a prompter combines the lessons of prior studies on checklists and prompting with leadership “best practices” [25], such as egalitarian leadership [26] and closed-loop communication [27].

Ideally, one of the existing team members should be able to play a role of prompter, without any added cost. However, in resource constraint situations, the team lead should focus on resuscitation and as soon as the time allows review the checklist to see if anything has been missed.

The CERTAIN approach was well received by the participants. However, it is worth noting that software usability limitations were evident in the post-intervention survey. With only 33% of participants feeling the software is easy to use, a combination of improved training and interface may be necessary to make this practical in real high-stress environments. In this simulation study, due to volunteers’ time and schedule constraints, the training was limited to 90 min. Most initial training in clinical resuscitation models (ACLS, BLS, or ATLS) are 12 h courses (usually two full training days) [28].

Another potential limitation could be that, even though the didactic sessions and practice scenarios were focused on tool usability rather than the scenario performance, having these done just prior to final testing could have influenced the performance.

The inferences from our results are further limited due to the simulation nature of this study, as well as the small sample size. Simulation training has been increasingly suggested as a valid research and training tool paired with good outcomes which makes it the ideal scenario to test a new method and clinical software [29]. The spectrum of scenarios encountered by our test subjects was limited to three common types: respiratory distress and hypoxia, hypotension due to severe sepsis/septic shock, and chest pain secondary to acute coronary syndrome. These cases were chosen as they are the most common clinical presentations in hospitalized medical patients [30–33]. Each clinician serving as his/her own control minimized the effect of variability in general medical knowledge. However, the absence of the control group of clinicians who were simply re-tested may limit the ability to discern the effectiveness of CERTAIN prompting vs training.

## Conclusion

Prompting with an electronic checklist (CERTAIN) improves clinical performance, subjective perceptions, and confidence of bedside clinicians confronted with typical emergency medical scenarios in high-fidelity simulation environment. Enhanced design/usability and better training are needed to leverage potential benefits of electronic checklist at the bedside of critically ill patients.

## Additional files

Additional file 1: Structure of the study design. (DOCX 46 kb)

Additional file 2: Example of standardized clinical scenario. (DOCX 99 kb)

Additional file 3: Satisfaction survey: a post-CERTAIN survey. (DOCX 33 kb)

Additional file 4: Snapshot of CERTAIN tool. (DOCX 122 kb)

## References

[1] Sebat F, Musthafa AA, Johnson D (2007). Effect of a rapid response system for patients in shock on time to treatment and mortality during 5 years*. Crit Care Med.

[2] Manor-Shulman O, Beyene J, Frndova H, Parshuram CS (2008). Quantifying the volume of documented clinical information in critical illness. J Crit Care.

[3] Woods DD, Patterson ES, Roth EM (2002). Can we ever escape from data overload? A cognitive systems diagnosis. Cogn Tech Work.

[4] Pickering BW, Hurley K, Marsh B (2009). Identification of patient information corruption in the intensive care unit: using a scoring tool to direct quality improvements in handover*. Crit Care Med.

[5] Gawande A (2010). The checklist manifesto: how to get things right. Vol 200: metropolitan books New York.

[6] Haynes AB, Weiser TG, Berry WR (2009). A surgical safety checklist to reduce morbidity and mortality in a global population. N Engl J Med.

[7] Weiss CH, Moazed F, McEvoy CA (2011). Prompting physicians to address a daily checklist and process of care and clinical outcomes: a single-site study. American Journal of Respiratory and CriticalCare Medicine.

[8] Kilickaya O, Bonneton B, Gajic O. Structured Approach to Early Recognition and Treatment of Acute Critical Illness In Annual Update in Intensive Care and Emergency Medicine. 2014;2014(2014):689–703. 10.1007/978-3-319-03746-2_51 edited by Jean-Louis Vincent.

[9] Rivers EP, Nguyen HB, Sepsis AD (2003). A landscape from the emergency department to the intensive care unit*. Crit Care Med.

[10] Kumar A, Zarychanski R, Light B (2010). Early combination antibiotic therapy yields improved survival compared with monotherapy in septic shock: a propensity-matched analysis*. Crit Care Med.

[11] De Luca G, Suryapranata H, Ottervanger JP, Antman EM (2004). Time delay to treatment and mortality in primary angioplasty for acute myocardial infarction. Circulation.

[12] Hacke W, Kaste M, Bluhmki E (2008). Thrombolysis with alteplase 3 to 4.5 hours after acute ischemic stroke. N Engl J Med.

[13] McAdams DJ. Acute hospitalist medicine and the rapid response system. Textbook of Rapid Response Systems 2011:47–53.

[14] Hales BM, Pronovost PJ (2006). The checklist––a tool for error management and performance improvement. J Crit Care.

[15] Eagle DM, Coltvet G, Farley D (2010). The Mayo Clinic, multidisciplinary simulation center. J Surg Educ.

[16] Arriaga AF, Bader AM, Wong JM (2013). Simulation-based trial of surgical-crisis checklists. N Engl J Med.

[17] Chan PS, Khalid A, Longmore LS, Berg RA, Kosiborod M, Spertus JA (2008). Hospital-wide code rates and mortality before and after implementation of a rapid response team. JAMA.

[18] Moretti MA, Cesar LAM, Nusbacher A, Kern KB, Timerman S, Ramires JAF (2007). Advanced cardiac life support training improves long-term survival from in-hospital cardiac arrest. Resuscitation.

[19] ATLS Subcommittee; American College of Surgeons’ Committee on Trauma; International ATLS working group. Advanced trauma life support (ATLS®): the ninth edition. J Trauma Acute Care Surg. 2013;74(5):1363-6. 10.1097/TA.0b013e31828b82f5. PubMed PMID: 23609291. 10.1097/TA.0b013e31828b82f5 23609291

[20] Smith KK, Gilcreast D, Pierce K (2008). Evaluation of staff’s retention of ACLS and BLS skills. Resuscitation.

[21] Low D, Clark N, Soar J (2011). A randomised control trial to determine if use of the iResus© application on a smart phone improves the performance of an advanced life support provider in a simulated medical emergency. Anaesthesia.

[22] Cooper S, Cant R, Porter J (2010). Rating medical emergency teamwork performance: development of the TEAM emergency assessment measure (TEAM). Resuscitation.

[23] Rivers EP, Nguyen HB, Huang DT, Donnino M (2004). Early goal-directed therapy. Crit Care Med.

[24] Cannon CP, Gibson CM, Lambrew CT (2000). Relationship of symptom-onset-to-balloon time and door-to-balloon time with mortality in patients undergoing angioplasty for acute myocardial infarction. JAMA.

[25] Havyer RD, Wingo MT, Comfere NI (2014). Teamwork assessment in internal medicine: a systematic review of validity evidence and outcomes. J Gen Intern Med.

[26] Calhoun AW, Boone MC, Porter MB, Miller KH (2014). Using simulation to address hierarchy-related errors in medical practice. Perm J.

[27] Härgestam M, Lindkvist M, Brulin C, Jacobsson M, Hultin M (2013). Communication in interdisciplinary teams: exploring closed-loop communication during in situ trauma team training. BMJ Open.

[28] Association AH. http://cpr.heart.org/AHAECC/CPRAndECC/Training/HealthcareProfessional/AdvancedCardiovascularLifeSupportACLS/UCM_473186_Advanced-Cardiovascular-Life-Support-ACLS.jsp.

[29] McLaughlin SA, Doezema D, Sklar DP (2002). Human simulation in emergency medicine training: a model curriculum. Acad Emerg Med.

[30] Seferian EG, Afessa B (2006). Demographic and clinical variation of adult intensive care unit utilization from a geographically defined population. Crit Care Med.

[31] Simpson H, Clancy M, Goldfrad C, Rowan K (2005). Admissions to intensive care units from emergency departments: a descriptive study. Emerg Med J.

[32] Staudinger T, Stoiser B, Müllner M (2000). Outcome and prognostic factors in critically ill cancer patients admitted to the intensive care unit. Crit Care Med.

[33] Knaus WA, Wagner DP, Zimmerman JE, Draper EA (1993). Variations in mortality and length of stay in intensive care units. Ann Intern Med.

