# Supplementary figures and images for: Prompting with electronic checklist improves clinician performance in medical emergencies: a high-fidelity simulation study
Source: Int J Emerg Med. 2018 Apr 27;11:26. doi: 10.1186/s12245-018-0185-8 (PMC5924513; doi:10.1186/s12245-018-0185-8)

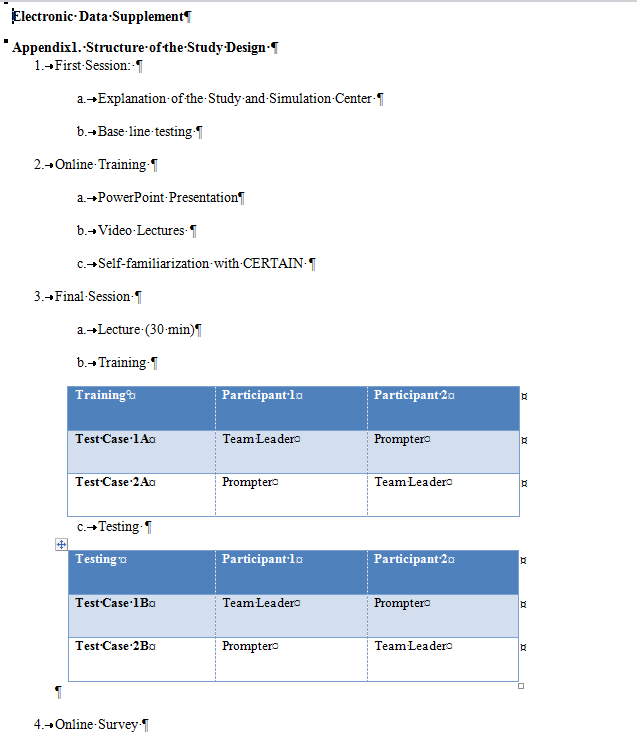

Supplement: Supplementary file 1 — Structure of the study design. (DOCX 46 kb) [file 12245_2018_185_MOESM1_ESM.docx]

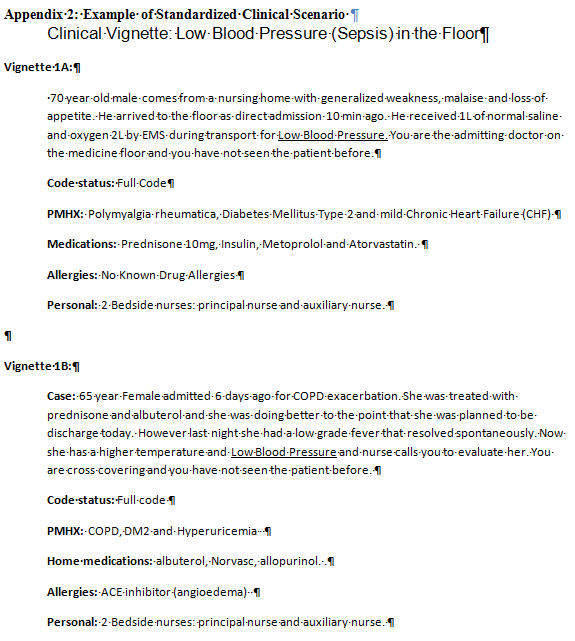


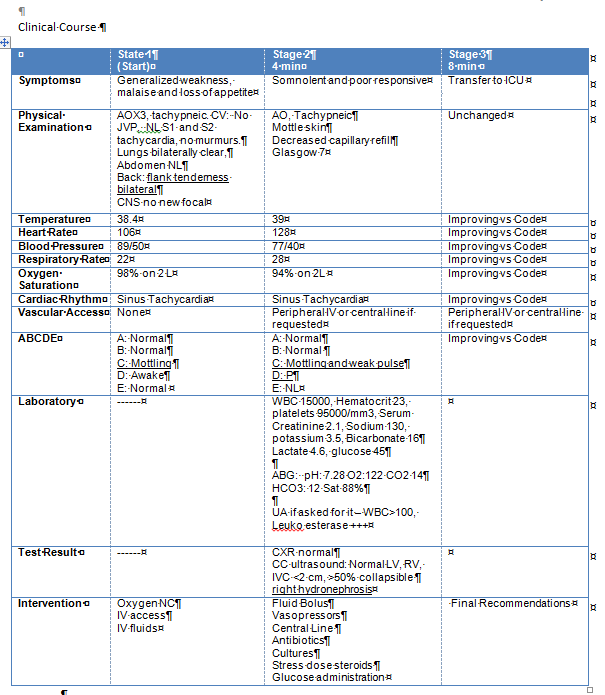

Supplement: Supplementary file 2 — Example of standardized clinical scenario. (DOCX 99 kb) [file 12245_2018_185_MOESM2_ESM.docx]

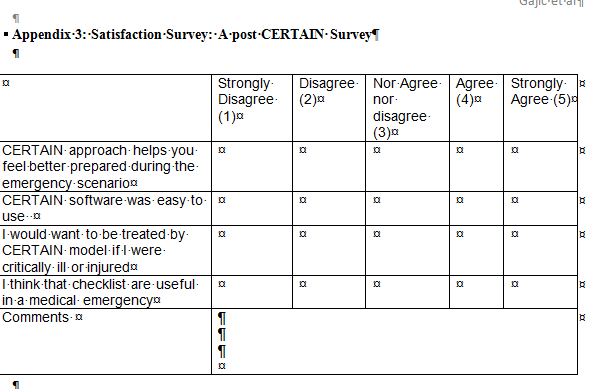

Supplement: Supplementary file 3 — Satisfaction survey: a post-CERTAIN survey. (DOCX 33 kb) [file 12245_2018_185_MOESM3_ESM.docx]

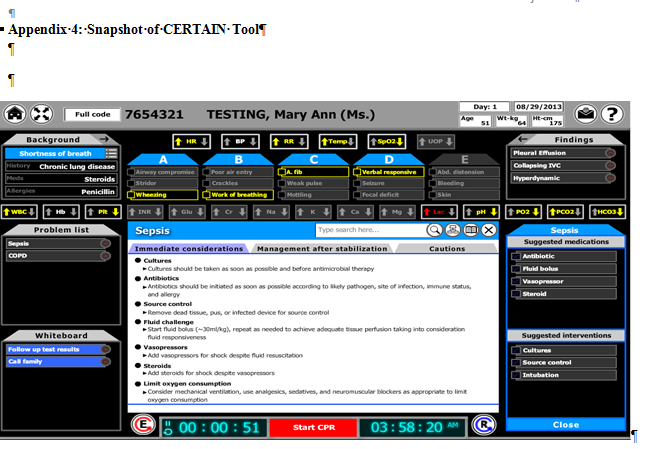

Supplement: Supplementary file 4 — Snapshot of CERTAIN tool. (DOCX 122 kb) [file 12245_2018_185_MOESM4_ESM.docx]
